# Eradication of specific donor-dependent variations of mesenchymal stem cells in immunomodulation to enhance therapeutic values

**DOI:** 10.1038/s41419-021-03644-5

**Published:** 2021-04-06

**Authors:** Chunxue Zhang, Liqiang Zhou, Zhen Wang, Wenxia Gao, Wei Chen, Huina Zhang, Bo Jing, Xu Zhu, Lei Chen, Changhong Zheng, Kaiyan Shi, Li Wu, Liming Cheng, Kunshan Zhang, Yi Eve Sun

**Affiliations:** 1 Stem Cell Translational Research Center, Tongji Hospital, Tongji University, School of Medicine, Shanghai, 200065 China; 2 Shanghai Institute of Stem Cell Research and Clinical Translation, Shanghai East Hospital, Tongji University, School of Medicine, Shanghai, 200120 China; 3 School of Life Science, Southern University of Science and Technology (SUSTech), Shenzhen, 518055 China; 4 Key Laboratory of Spine and Spinal Cord Injury Repair and Regeneration (Tongji University), Ministry of Education, Shanghai, 200065 China

**Keywords:** Neuroimmunology, Mesenchymal stem cells, Stem-cell research

## Abstract

Mesenchymal stem cells (MSCs) are one of the most widely clinically trialed stem cells, due to their abilities to differentiate into multiple cell lineages, to secrete regenerative/rejuvenative factors, and to modulate immune functions, among others. In this study, we analyzed human umbilical-cord-derived MSCs from 32 donors and revealed donor-dependent variations in two non-correlated properties, (1) cell proliferation, and (2) immune modulatory functions in vitro and in vivo, which might explain inconsistent clinical efficacies of MSCs. Through unbiased transcriptomic analyses, we discovered that IFN-γ and NF-κB signaling were positively associated with immune modulatory function of MSCs. Activation of these two pathways via IFN-γ and TNF-α treatment eradicated donor-dependent variations. Additional transcriptomic analyses revealed that treatment with these two factors, while having abolished donor-dependent variations in immune modulatory function, did not overall make different donor-derived MSCs the same at whole transcriptomic levels, demonstrating that the cells were still different in many other biological perspectives, and may not perform equally for therapeutic purposes other than immune modulation. Pre-selection or pre-treatment to eradicate MSC variations in a disease-treatment-specific manner would therefore be necessary to ensure clinical efficacies. Together this study provided novel insights into the quality control perspective of using different-donor-derived MSCs to treat inflammation-related clinical conditions and/or autoimmune diseases.

## Introduction

Mesenchymal stem cells (MSCs) could self-renew and differentiate into three typical MSC lineages, i.e., osteocytes, chondrocytes, and adipocytes^1^, in addition to other atypical lineages such as endothelial cells^2^, muscle cells^3^, neural lineage cells^4^, etc. MSCs are known to exist in many different tissues including bone marrow, adipose tissues, dental pulp, umbilical cord, etc^5^., and played critical roles in modulating tissue microenvironment by secreting growth factors, promoting vasculature development, and antagonizing inflammatory immune functions^6^. Due to easy accessibility and expansion in culture, MSCs from different tissue sources have been selected as potential therapeutic agents to repair bone, cartilage, as well as treat autoimmune diseases and chronic disorders involving tissue inflammation such as heart failure, diabetes, and stroke^5^, as well as neurodegenerative disorders including Alzheimer’s and Parkinson’s diseases^7^.

The classic method to define MSCs from various tissue sources are the usage of cell-surface markers, i.e., all MSCs are positive for CD44, CD73, CD90, CD105 and negative for CD45, CD19, CD11b, CD34, HLA-DR, as well as their potential to spontaneously differentiate into bone, cartilage, and fat cells^1^. It is increasingly acknowledged that huge diversities and heterogeneities are associated with MSCs. First, MSCs from different tissues are different, as indicated by dramatic differences in their transcriptomes (Supplementary Fig. S1), and the differentially expressed genes are associated with cell adhesion, cell proliferation, cytokine signaling, wound healing, and organ development, all of which are critical biological features (Supplementary Fig. S1). This finding suggests that MSCs from different tissue sources might be suitable for different therapeutic purposes. Secondly, our previous studies had demonstrated that MSCs derived from umbilical cord obtained from the same donor showed heterogeneities in that certain MSC clones did and others did not express a neural lineage marker gene, *NESTIN*, and these different clones had different immune suppressive features (Supplementary Fig. S2). Lastly, donor-dependent variations of MSCs have also been documented including their angiogenic abilities and chondrogenic potentials^8,9^. These variations are strong confounding factors regarding MSC-based therapies and, without recognizing these variations, may result in variable clinical outcomes that could impede clinical translation of MSC-based interventions.

MSCs could suppress proliferation and activation of many immune cells including T cells, B cells, NK cells, as well as microglia. Microglia maintain homeostasis of immune microenvironment of the central nervous system (CNS)^10,11^ and play critical roles in many neurological diseases involving neuroinflammation, including stroke^12^, traumatic brain injury^13^, Parkinson’s disease^14^ and spinal cord injury^15^. By suppressing microglia activation and secreting various factors including miRNAs^16^, cytokines^17^, chemokines^18^, as well as growth factors^19^, MSC-based therapies have been proposed to develop therapies for a number of neurological disorders^20–22^. However, the aforementioned MSC variations would potentially affect clinical trial outcomes.

In this study, we analyzed human umbilical-cord-derived MSCs from 32 donors for their proliferation rates as well as suppressive function on BV2 microglial cells and revealed substantial variations. In addition, we used lipo-poly-saccharide (LPS)-injection-induced neural inflammation animal model to evaluate immune-suppressive function of MSCs in vivo, and obtained consistent in vitro/in vivo results. Transcriptomic analyses of different MSC lines revealed differences in IFN-γ and NF-κB signaling, which correlated well with immune modulatory function. Activation of IFN-γ and NF-κB signaling by treating MSCs with IFN-γ and TNF-α, normalized weak and strong MSC lines, and overall enhanced their function in immune modulation both in vitro and in vivo, suggesting that pretreatment of MSCs might provide more consistent clinical outcomes in treating CNS injuries or autoimmune diseases.

Further transcriptomic analyses of MSCs with and without 2-factor treatment revealed that while treatment normalized immune regulatory functions of different MSC lines, the overall transcriptome of MSCs after treatment did not become the same, indicating that other differences in biological features of different MSC lines still exist even after 2-factor treatment. Therefore, if, for example, MSCs would be used for treating osteoporosis or limb ischemia^23,24^, their osteogenic or angiogenic function would be critical, donor-dependent variations in MSC treatment efficacies likely will not be eliminated by 2-factor treatment, but by other means. Together, preselection or pretreatment to eradicate MSC variations in a disease-treatment-specific manner would therefore be necessary to ensure clinical efficacies.

## Results

### Donor-dependent variation of human umbilical cord-derived MSC in proliferation and immune modulation

**Fig. 1:**
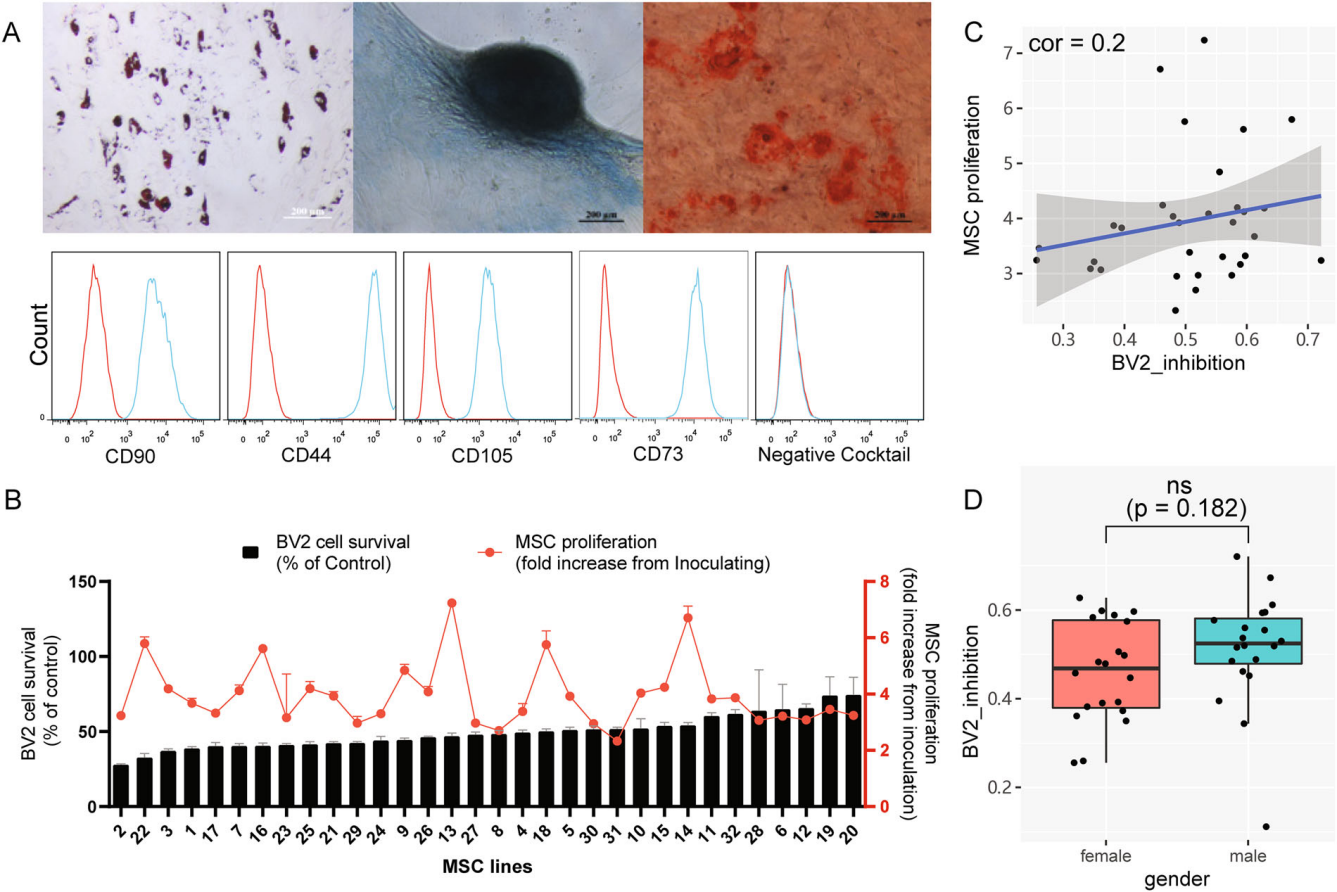
Donor-dependent variations of huMSC proliferation and immune modulation. **A** Three lineage differentiation of huMSCs and expression of cell surface markers. Upper Panel from left to right: MSCs differentiated to adipocytes, chondrocytes, and osteocytes. **B** BV2-suppression capacity and proliferation capacity of 32 lines of huMSCs. The black bars represent the ratio of BV2 cell mass after 48 h cultured in MSC-CM compared to RPMI-1640 (control medium). The red dots represent the ratio of total huMSC mass at 72 h after inoculation compared to 1 h after inoculation. **C** correlation between BV2-suppression capacity and huMSC proliferation capacity of 32 huMSC lines. **D** Boxplot shows BV2-suppression capacity between male (*n* = 20) and female (*n* = 20) huMSC lines, eight extra huMSC lines were added to increase the statistical power.

Cultured human umbilical-cord-derived MSC (huMSC) from 32 donors at passage 5 (P5) were characterized by their ability to differentiate into bone, cartilage, and fat cells (Fig. 1A), as well as carrying cell surface markers CD 44, 73, 90, 105, but not CD19, 34, 45, 11b, or HLA-DR (Fig. 1A), which were basic common features for MSCs. However, these 32 lines of P5 MSCs demonstrated variability in their proliferation rates (Fig. 1B and Supplementary Table S2). To investigate whether immunomodulation function of these 32 different MSC lines were also variable, we first used an in vitro culture model, where a mouse microglial cell line BV2 cells were cultured for 48 h in conditioned medium (CM) collected from 32 different lines of P5 huMSCs. The 32 lines of huMSCs were inoculated at the same density and cultured for 72 h for collection of CM. The suppressive effect of CM from each line of huMSCs on BV2 was deemed as an index for capability of microglia/immune suppression (Fig. 1B). Results showed that the suppressive indices (SI) of MSCs from different donors were variable, ranging from 0.256 to 0.721 (Fig. 1B). We further determined correlations between huMSC proliferation rates and their BV2 immune suppressive indices as well as potential influences from gender to immune modulation function of huMSCs, and found that neither proliferation rate nor gender appeared to significantly influence the ability of huMSCs to suppress BV2 cells (Fig. 1C, D).

### Donor-dependent variations of huMSC immunomodulation in vivo

**Fig. 2:**
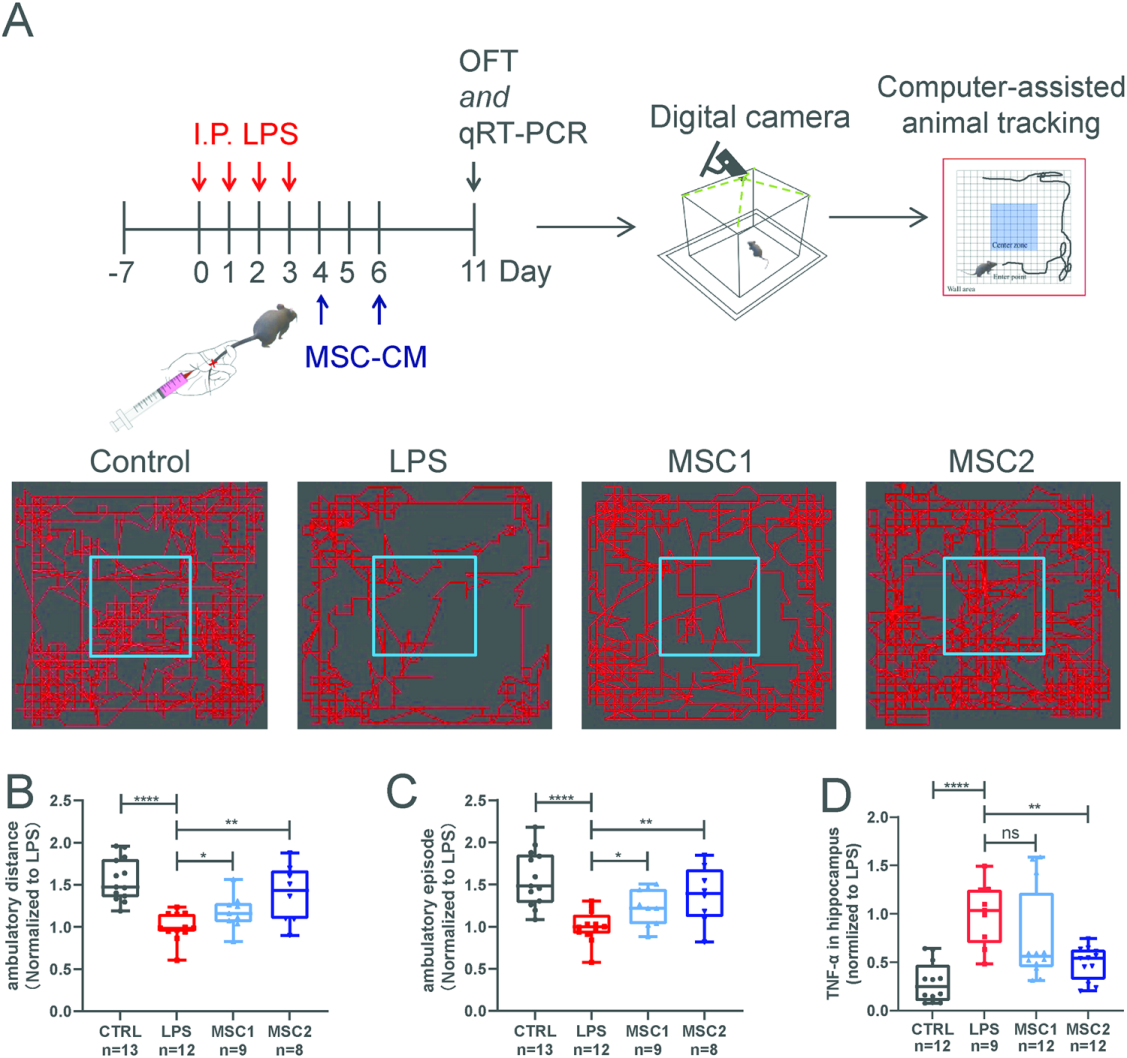
Donor-dependent variations in immune modulation of huMSCs in LPS-induced neural inflammation animal model in vivo. **A** Schematic diagram shows in vivo assessment MSC-CM treatment efficacies in LPS-induced neuroinflammation animal model. Open-field-test was used as the main behavioral readout. Boxplots show ambulatory distance (**B**) and episodes (**C**) of the open-field-test of each treatment groups. All data were normalized to the average value of the LPS group. **D** Boxplot shows inflammatory factors *TNF-α* mRNA levels in hippocampus of each group. Data were also normalized to the average value of the LPS group, and presented as mean ± SEM, *P < 0.05, **P < 0.01, ****P < 0.0001.

To investigate the effect of donor-dependent differences on potential clinical efficacy of MSCs, we selected two lines of huMSC with immune SI equal to 0.35 (MSC1) and 0.67 (MSC2), respectively. We established a mouse model of intra-peritoneal injection of LPS-induced neuroinflammation^25^ (Fig. 2A). After daily injection of LPS at a dose of 1 mg/kg, mice started to develop a series of abnormalities including lethargy (sleepiness and immobility), arched back (huddling), hair bristling (piloerection), ptosis, weight loss, and anal-rectal dysfunction. On the 4^th^ and 6^th^ days after the first LPS injection, mice were treated with CM from MSC1 and MSC2 via tail vein injections (Fig. 2A). Open field tests were performed on the 11^th^ day to access neural deficits. Immobility after LPS treatment was obvious and could be quantitatively reflected by reduced ambulatory distance and episodes (Fig. 2B, C). Neural inflammation could be reflected by increased hippocampal *TNF-α* mRNA levels (Fig. 2D). Tail vein injection of CM from MSC2 (SI = 0.67) reduced neural inflammation and improved animal motor behavior (Fig. 2B–D), whereas CM from MSC1 (SI = 0.35) only showed weaker or a trend (without statistical significance) of improvement (Fig. 2B–D). Similarly, in a mouse crush injury-based spinal cord injury model, tail vein transfusion of huMSC-A with SI = 0.67 at a dose of 1 million cells/animal (weighed about 25 g) at day three post-surgery also improved animals’ walking behavior (by BMS scoring) (Supplementary Fig. S3). However, huMSC-C and B lines with SI = 0.26 and 0.52, respectively, did not significantly improve animal behavior (Supplementary Fig. S3). Together, these data suggested that donor-dependent variations of huMSCs in immunomodulation would likely affect their therapeutic efficacies, when treating neural-inflammation-related clinical conditions.

### Transcriptomic analysis of huMSC showed positive correlations of IFN-γ and NF-κB signaling with immune modulatory function

**Fig. 3:**
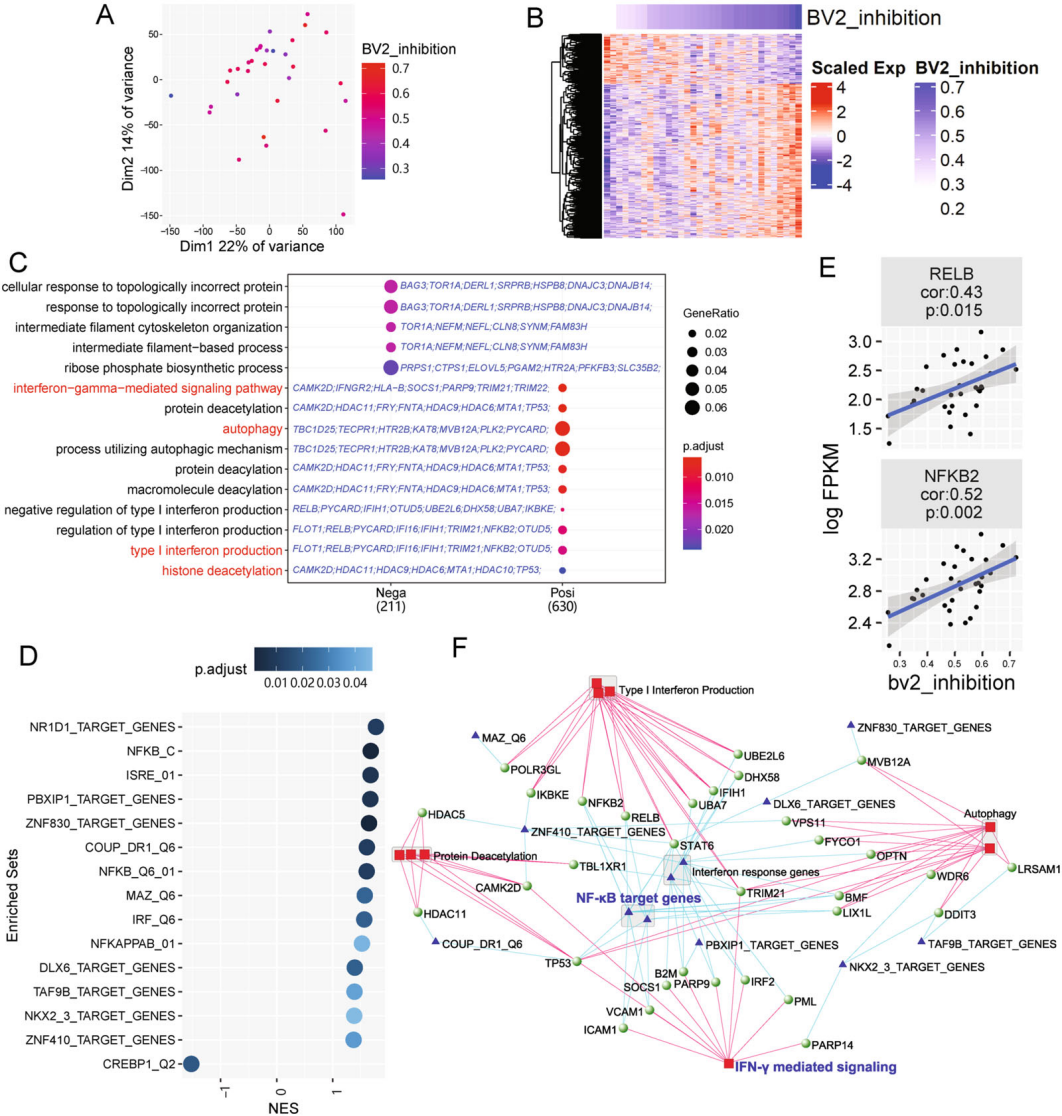
Transcriptomic analysis of 32 lines of huMSCs revealed functional modules related to BV2-suppression capacity. **A** PCA of 32 huMSC lines. **B** Heatmap shows expression and clustering of genes, expression of which significantly correlated to BV2 inhibition capacity of 32 lines of huMSCs. **C** GO enrichment analysis of BV2 inhibition correlated genes. IFN-γ, autophagy, and related terms are highlighted in red, which are positively correlated with BV2 inhibition. Candidate genes belonging to each GO term are shown in blue. **D** GSEA analysis of enriched TFBS targeting gene sets, most of which, except for one, were positively correlated with BV2 inhibition (normalized enrichment score (NES) > 0). **E** Correlation between gene expression levels of two subunits of NF-κB complex and BV2 inhibition capacity. **F** Network shows connection between enriched GO terms, TFBS targeting gene sets, and positively correlated genes. Red squares represent GO terms and blue triangles represent TFBS targeting gene sets. The nodes of green balls are genes, which belonging to both corresponding GO terms and TFBS targeting gene sets.

To reveal molecular mechanisms underlying the immune suppressive function of huMSCs, we analyzed the transcriptome of all 32 lines of MSCs. Two-Dimensional Principle Component Analysis (PCA) showed MSC whole transcriptomes were dispersed, and not correlated well with BV2-suppressive function (Fig. 3A). We later isolated 1037 genes that show significant positive and negative correlations (784 positively correlated genes and 253 negatively correlated genes) with BV2 inhibition levels (Fig. 3B).

Gene ontology (GO) enrichment analysis showed that genes positively correlated with BV2 inhibition levels were linked to functions including “autophagy”, “interferon-γ mediated signaling pathway” and “histone deacetylation”, meanwhile genes negatively correlated with BV2 inhibition levels were related to “response to topologically incorrect protein” and “intermediate filament organization” (Fig. 3C and Supplementary Table S3). A network analysis based on GO semantic similarity also supported these results (Supplementary Fig. S4). Additionally, we analyzed transcription factor binding site (TFBS)-enriched gene sets in our data, results showed that both NF-κB related TFBS (NFKB_C, NFKB_Q6_01, NFKAPPAB_01) and interferon response TFBS (ISRE_01, IRF_Q6) were present in genes that were positively correlated with BV2 inhibition (Fig. 3D), indicating that huMSC-intrinsic expression levels of NF-κB signaling pathway and IFN-γ signaling pathway reflected immunomodulation capacities of huMSCs. Indeed, NF-κB subunits *RELB* and *NFKB2* showed positive correlations with BV2 inhibition levels (Fig. 3E).

Further construction of a regulation network using genes positively correlated with BV2-cell inhibition, together with their GO terms and TF-binding, illustrated a more comprehensive network related to immunomodulation capacity of MSCs. The gene regulation network revealed many NF-κB pathway related genes, such as *NFKB2*, *RELB*, *IKBKE*, as well as IFN-γ signaling pathway genes. In fact, it has been reported that without stimulation of IFN-γ, MSCs showed very limited immunomodulation effect, and blockade of IFN-γ or IFN-γR impaired immunomodulation function of MSCs^26,27^. Interestingly, histone deacetylation as well as autophagy, the other two GO themes linked to BV2-cell inhibition, appeared to be also well connected to NF-κB and IFN-γ signaling (Fig. 3F)

### Pretreatment with IFN-γ and TNF-α eliminated donor-dependent variations of huMSC in immunomodulation

Transcriptomic analysis suggested that differences in the expression of genes related to the IFN-γ and NF-κB signaling pathway, which could be activated by TNF-α, may be a critical reason for donor-dependent variations in huMSCs regarding immune modulation. It has also been well-known that inflammatory factors such as IFN-γ and TNF-α could enhance the immunomodulatory capacity of MSCs, and several studies have reported improvement of treatment efficacies of MSCs by pretreatment with inflammatory factors^28–30^.

**Fig. 4:**
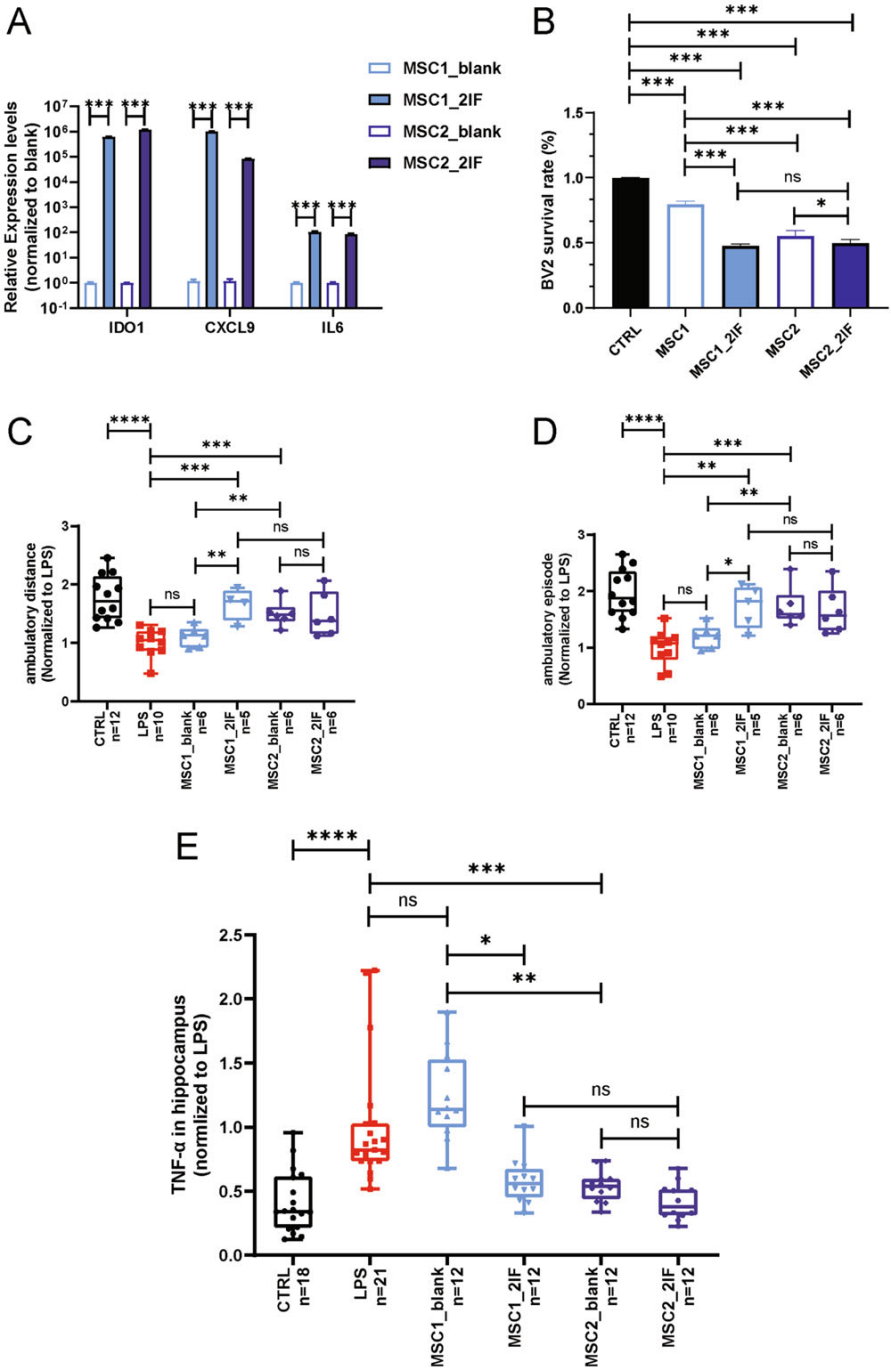
Two-factor stimulation abolished donor-dependent variations of huMSCs in immune suppression. **A** Anti-inflammation genes *IDO1*, *CXCL9* and *IL6* were extensively up-regulated in huMSCs by stimulation of TNF-α and IFN-γ (2-factors). **B** In vitro analysis on BV2 inhibition showed elimination of donor-dependent variations in immune-modulations by two factors. **C**–**E** eradication of specific donor-dependent variations of huMSCs regarding treatment efficacies in LPS-induced neuroinflammation model, by open-field-motor scoring and hippocampal *TNF-α* expression. All data were normalized to LPS group, and were presented as mean ± SEM, *p < 0.05, **p < 0.01, ***p < 0.001, ****p < 0.0001.

We therefore decided to use the two factors (IFN-γ and TNF-α) to stimulate two MSC lines with SI equaled to 0.67 and 0.26, respectively, to determine whether line to line variations could be eradicated. As described in our previous work^31^, key anti-inflammatory factors *IDO1*, *CXCL9*, and *IL6* were extensively upregulated after 2-factor stimulation (Fig. 4A). BV2-cell inhibition assays showed that the inhibition capacities of MSC1 and MSC2 were significantly increased after 2-factor stimulation and differences between the two lines were eliminated (Fig. 4B). The in vivo treatment efficacies of the two MSC lines were then examined, before and after stimulation, using the LPS-induced neural inflammation mouse model. As expected, MSC2 showed better therapeutic effect than MSC1 without 2-factor stimulation. However, after stimulation, the therapeutic efficacy of MSC1 was no longer statistically different from that of MSC2 (Fig. 4C–E). These results suggest that 2-factor stimulation may improve the anti-inflammatory function of huMSCs, and eradicate the donor-dependent variations.

### IFN-γ and TNF-α stimulation of huMSCs enhanced immunomodulation but suppressed proliferation

**Fig. 5:**
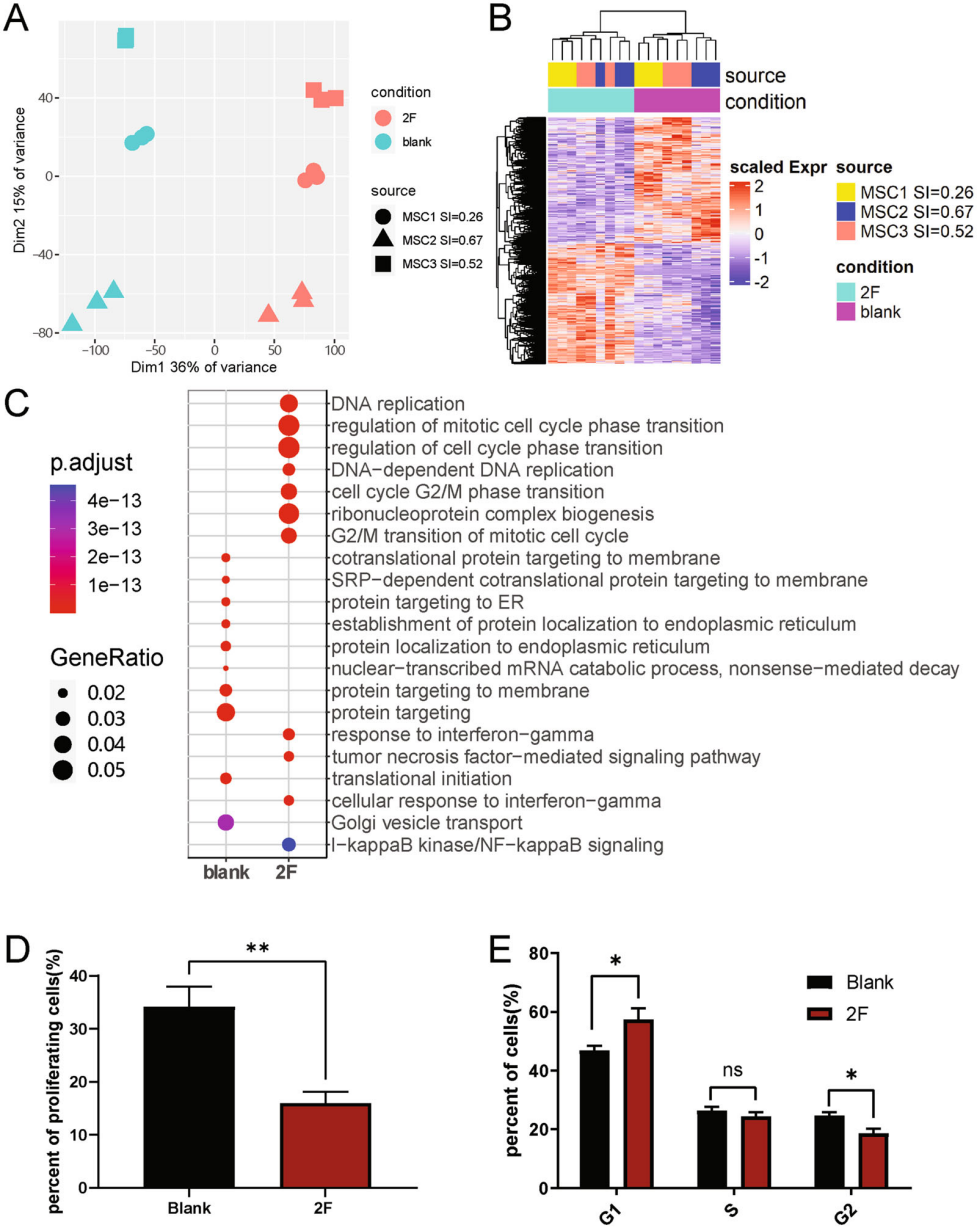
Transcriptome analysis of huMSCs before and after 2-factor treatment. **A** PCA of all samples including 3 different genetic background and 2 conditions. **B** Heatmap demonstrating differentially expressed genes (DEGs) between 2 conditions. **C** GO enrichment analysis of DEGs in B. **D** Bar-plot demonstrating percentage of proliferating cells before and after 2-factor stimulation based on EdU assay. **E** Bar-plot shows percentage of cells in each cycle phase before and after cytokine stimulation based on flow cytometry.

To reveal whether inflammatory factor stimulation could mobilize the intrinsic regulatory network of MSCs to enable global enhancement of immunomodulation, and thus make the different huMSC lines overall more similar, we analyzed the transcriptomes of huMSCs before and after stimulation. PCA results showed that inflammatory factor stimulation dramatically altered the transcriptomes of huMSCs (Fig. 5A), but the three different huMSC lines were still different at whole transcriptomic levels. We identified 9702 differentially expressed genes (4724 upregulated and 4978 downregulated) before and after stimulation (Fig. 5B). Interestingly, the functional enrichment analysis showed that among the genes that were significantly upregulated after stimulation, the greatest enrichment was in DNA replication and cell cycle-related functions (Fig. 5C and Supplementary Table S4). A network analysis based on the semantic similarity of GO terms revealed that genes that were upregulated after stimulation were concentrated in five functional modules: DNA replication and cell cycle, response to IFN-γ and TNF-α signals, antigen processing and presentation, macromolecular translocation, macromolecular synthesis, and degradation (Supplementary Fig. S5). On the other hand, there were four functional modules enriched in genes downregulated by 2-factors: TGF-β and SMAD signaling pathways, cell differentiation, vesicle assembly, and macromolecular metabolic process (Supplementary Fig. S6).

To verify the results of the transcriptome analysis, we examined the proliferative capacity of huMSCs before and after stimulation and found a significant decrease in the number of proliferating cells after 2-factor stimulation (Fig. 5D). In addition, cell cycle analysis indicated that inflammatory factor stimulation retained huMSCs in the G1 phase and consequently reduced the number of cells in the G2 phase (Fig. 5E). This confirmed the transcriptomic results.

**Fig. 6:**
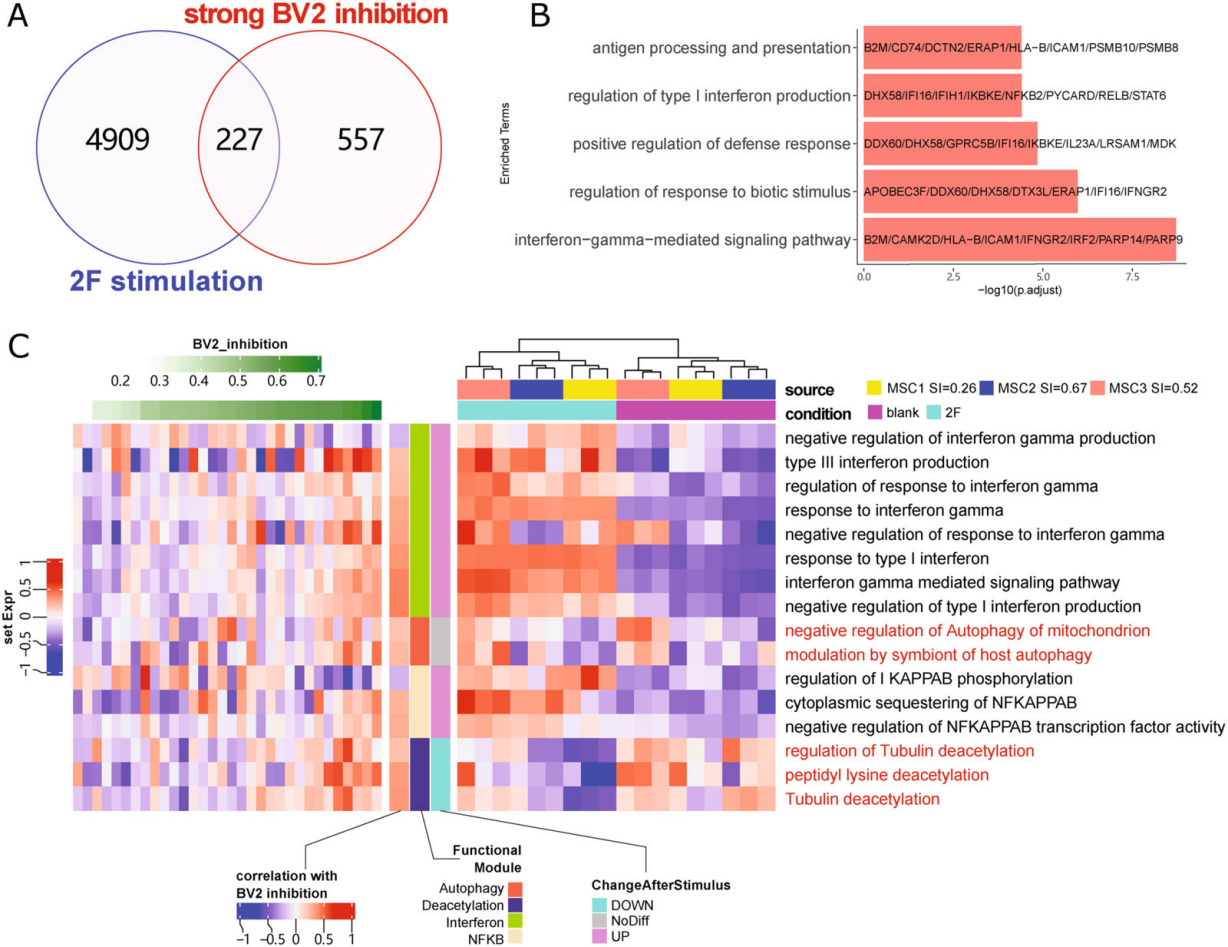
Two transcriptomic data sets (32 huMSC lines and 3 huMSC lines with and without 2-factor stimulation) revealed common genes and pathways. **A** Venn diagram shows the overlap between genes up-regulated by 2-factor stimulation and genes positively correlated to BV2 inhibition capacity. **B** GO enrichment analysis of overlapped 227 genes. **C** GSVA heatmap shows specific GO-term associated gene sets in 32 huMSCs that positively or negatively correlated with BV2-cell immune suppressive capacity, and their expression levels before and after 2-factor stimulation. Results clearly indicated that interferon and NF-κB pathway associated genes were upregulated upon 2-factor stimulation, whereas genes related to autophagy and protein deacetylation did not correlate well with 2-factor stimulation.

We further analyzed the 227 genes, which were both upregulated by 2-factor stimulation and were positively correlated with capacities of BV2-cell inhibition (Fig. 6A, B). Function of these genes focused on immune related pathways included IFN-γ signaling pathway, Type I interferon production and antigen processing. Interestingly, based on gene set variation analysis (GSVA), genes linked to “protein deacetylation” and “autophagy”, while positively correlated with BV2 immune suppressive capacities of MSCs, were not upregulated upon 2-factor treatment (Fig. 6C and Supplementary Table S5). These results suggested that 2-factor stimulation, while potentially enhancing the immunomodulatory function of huMSCs by activating IFN-γ and NF-κB signaling pathways and eradicating donor-dependent variations of huMSCs, also made huMSCs proliferate much slower. Whether or not this is a desired feature for MSC-based therapeutic interventions would depend on the detailed therapeutic targets/purposes.

## Discussion

Many neurological disorders including spinal cord injury, stroke, Alzheimer’s and Parkinson’s diseases involve neural inflammation. MSC-based therapies were designed to inhibit the proliferation and activation of inflammatory immune cells including microglia, thereby promoting CNS repair or preventing deleterious progressions. Our study revealed donor-dependent variations of huMSCs in immune suppression, which was a critical message to inform the various MSC-based clinical trial studies that not all MSCs were equal and preselection of suitable MSCs would be key to produce effective clinical outcomes.

Transcriptomic analyses of different lines of huMSCs were effective to identify molecular biological properties of MSCs underlying variations in their efficacies. Such analyses enable the means to design pretreatments for different lines of huMSCs to normalize their function. While we were able to use 2-factor treatment to eradicate donor-dependent variations of huMSCs with respect to their immune suppressive function, 2-factor treatment did not abolish other differences between MSC lines from different donors. Although one of the major therapeutic functions of MSCs is immune modulation, MSCs have also been proposed to repair bone and cartilage, to promote regeneration of the vasculature, and to repair ischemic heart tissue, where the ability of MSCs to differentiate into chondrocytes and osteocytes, or secrete angiogenic factors and other trophic factors might be important. For those clinical applications, donor-dependent variation of MSCs likely also exist, and may also be quite different from how they vary in their immune modulatory function. For example, one MSC line suitable for treating liver cirrhosis may not be ideal to treat osteoporosis. Therefore, specific therapeutic purpose-based assays should be developed and used to address potential donor-dependent variations in treatment efficacies, and different means for normalization of treatment efficacies might need to be developed. Towards this line of research, trait-transcriptome association analysis as done in this study, could be quite useful.

Transcriptomic analyses and later biological validation studies indicated that 2-factor treatment of huMSCs, while enhancing immune suppressive function of huMSCs, and thus eradicating donor-dependent variations in immune modulation, also slowed huMSC proliferation. Therefore, 2-factor treatment should be avoided during the expansion period of huMSCs. Perhaps, such treatments should apply only during the last passage, prior to the cells being harvested for treatment. Whether pretreatment is an optimal approach or pre-selection is a better choice still remains to be determined, and must be determined in a disease-treatment-specific manner. Altogether, our study raised and solved an important issue regarding the quality control perspective of MSC products and potentially paved a new path toward success in MSC-based therapeutic interventions.

## Materials and methods

### Isolation and expansion of huMSC

HuMSC were isolated from different human umbilical cord (*n* = 32) samples following normal cesarean birth. Briefly, the blood vessels of the umbilical cord were removed to retain Wharton’s jelly. Wharton’s jelly was cut into 1 mm^3^ pieces, and then collagenase was used to digest the umbilical cord for 1 h at 37 °C. MSCs were cultured in α-MEM supplemented with 5% UltraGRO™ (Helios). Cultures were maintained at 37 °C with saturated humidity and 5% CO2. After 48 h, nonadherent cells were removed by washing, and media were changed every three days. MSCs were subcultured at 80% confluence following treatment with 0.25% trypsin-EDTA (Gibco) for 3 min at 37 °C. Cells were washed and harvested by centrifugation at 400 *g* for 3 min, then replated at a lower density (1000 cells/cm^2^) for additional expansion.

### Characterization of huMSC

#### FACS analysis

To verify the characteristics of MSCs, MSCs were treated with 0.25% trypsin-EDTA for 3 min at 37 °C and then harvested by centrifugation at 400 *g* for 3 min. Cell suspensions were washed twice with PBS and then incubated with antibodies from Human MSC Analysis Kit (BD Biosciences) and protected from light for 30 min at room temperature. Following incubation, cells were washed three times with PBS. The fluorescent intensity of the cells was evaluated using a flow cytometer (FACScan; BD Biosciences) and the data were analyzed using the FlowJo 7.6.1 software.

#### Differentiation of huMSC into adipogenic, osteogenic, and chondrogenic lineages

HuMSC were seeded into 24-well plate at 3 × 10^4^ cells/well and incubated in normal MSC growth medium at 37 °C in a humidified atmosphere with 5% CO_2_. Adipogenic, osteogenic, and chondrogenic differentiation media (Invitrogen) were used to exchange the medium once the cells reaching 80–100% confluence. The cultures were exchanged every three days. After 7–14 days of induction, adipogenesis was determined by staining with Oil Red O for intracellular lipid accumulation. After 21–28 days of induction, osteogenesis was visualized with Alizarin Red S staining, which was specific for calcium, and chondrogenic differentiation was stained with toluidine blue following standard procedures.

### Pretreatment of huMSC with IFN-γ and TNF-α

HuMSC were digested with trypsin-EDTA once cells reach 100% confluence. Cells were seeded into T75 cell culture flasks at a density of 1 × 10^6^ cells/T75. After 12 h, the medium was exchanged with fresh MSC complete medium including IFN-γ (10 ng/ml) and TNF-α (10 ng/ml). After 24-h incubation, the cells were washed three times with PBS and then cultured with fresh MSC medium for another 24 h.

### Collection of huMSC-conditioned medium (huMSC-CM)

HuMSC at passage 4 (P4) were digested with 0.25% trypsin-EDTA once cells reach 100% confluence. HuMSC were washed once with PBS and then seeded into T75 cell culture flasks at the same density (1 × 10^6^ cells/T75). After 72 h, the medium was collected into a 15 ml centrifuge tube and centrifuged at 2000 rpm for 10 min to discard the cells and cell debris. Supernatant was transferred to a new 15 ml centrifuge tube and then aliquoted into smaller volume unit for storage at −80 °C.

### Immunomodulatory properties of huMSC in vitro (BV2 proliferation assay)

The mouse microglia cell line BV2 was purchased from OBiO Technology (Shanghai) Corp. BV2 cells were cultured in RPMI-1640 supplemented with 20% FBS (Gibco) at 37 °C with saturated humidity and 5% CO_2_. Cells were plated into 96 well plate at density of 5 × 10^3^ cells/well in 100 µl complete medium, then incubated for 48 h, before switching to 100 µl complete medium (control) or huMSC-CM. After another 48 h of incubation, 10 µl CCK8 was added into every well and incubate for 4 h. OD value was measured at 450 nanometer using SpectraMax^®^ i3x (Molecular Devices). BV2 survival rate was analyzed by GraphPad Prism (version 8). To overcame random error, we repeat the assay for seven times. The average suppression effect (1- (BV2 survived in huMSC-CM)/(BV2 survived in RMPI-1640)) of seven repetitions was used as SI of each MSC line. All cells used in this study were tested as negative for mycoplasma contamination.

### Quantitative real-time polymerase chain reaction (qRT-PCR)

Total RNA was extracted from cultured cells or dissected tissues using Trizol reagent (Invitrogen). Equal amount of total RNA was subjected to reverse transcription using PrimeScript™ RT reagent Kit with gDNA Eraser (Takara) as instructed. Real-time PCR was performed on a Q5 real-time PCR system (Applied Biosystems) by using SYBR Premix EX Taq with ROX (Takara). Amplification results were normalized on the basis of the GAPDH mRNA levels in each sample. For real-time PCR, primers used for gene expression were listed in Supplementary Table S1.

### Transcriptomic analysis

RNA samples from cultured huMSC were sequenced using a standard Illumina protocol (Novogene Co., Ltd.). Reads were mapped to a human genome (hg19) by using hisat2 (v2.1). Gene counts and FPKM were estimated by HTSeq (v0.11) and StringTie (v1.3.5), respectively. Genes with FPKM > 1 in half of samples (16 of 32) were deemed as real expressed genes and used for correlation analysis, significantly correlated genes (*p* < 0.01, Student *T*-test) were applied to function analysis. The function *corAndPvalue* in *WGCNA* package was used to perform correlation analysis. The R package *clusterProfiler* was applied to perform GO enrichment analysis and GSEA analysis^32^. TFBS targeting gene sets were download from MSigDB (c3.TFT). DEG analysis was performed by R package *DESeq2*^33^. GO-terms Semantic Similarity network was constructed by R package *GOSemSim*^34^. Gene set variation analysis was performed by *GSVA* package^35^.

The FASTQ files have been deposited in the NCBI GEO database under accession number GSE165811.

### Animals

6–8 weeks male (20–23 g) C57BL/6NCrl mice were purchased from Beijing Vital River Laboratory Animal Technology (Beijing, China). All animals were raised in a specific pathogen-free (SPF) facility, under a 12–12 h day-night illumination cycle. Animals that fail to reach the endpoint of the experiment will be excluded. All animal were randomly grouped by investigator. The group allocation, conditioned medium injection and assessment of outcome were performed by different person in blind. All experimental procedures were complied with international guidelines for the care and use of laboratory animals and approved by the Animal Ethics Committee of Tongji University, Shanghai, China (Approval Number: TJAA06621102).

### LPS model

LPS (sigma) was administered to induce systemic inflammation. Single daily intraperitoneal (i.p.) injections of LPS (1 mg/kg) were repeated for 4 days. Control mice received i.p. saline injections under the same dosing schedule. The concentrated huMSC-CM was intravenous injected (200 µl/animal) on day 4 and day 6, respectively. Open field test was performed on day 11 after LPS injection. Mice were placed in a square arena (27.6 × 27.6 cm^2^) for 5 min. The distance (cm) traveled and episodes were analyzed using Activity Monitor 7 ENV-256T software.

### Statistical analysis

Data are presented as mean ± SEM. Statistical significance was assessed by unpaired two-tailed Student’s *t*-test. Student’s *t*-test was used to analyze data between two groups. All experiments were repeated at least three times. In all tests, *P* < 0.05 was considered a statistically significant difference between the mean values.

## Supplementary information

Supplementary information

Figure S1

Figure S2

Figure S3

Figure S4

Figure S5

Figure S6

Table S2

Table S3

Table S4

Table S5

## Data Availability

All experimental data are available in the main text or the supplementary materials.
