# Supplementary material for: Eradication of specific donor-dependent variations of mesenchymal stem cells in immunomodulation to enhance therapeutic values
Source: Cell Death Dis. 2021 Apr 6;12(4):357. doi: 10.1038/s41419-021-03644-5 (PMC8024246; doi:10.1038/s41419-021-03644-5)
Supplement: Supplementary file 1 — Supplementary information [file 41419_2021_3644_MOESM1_ESM.docx]

**Supplementary information**

**Materials and Methods**

**Immunomodulatory properties of huMSC in vitro (PBMC proliferation assay)**

Human PBMCs were isolated from fresh whole blood using Ficoll (GE Healthcare) with informed consent from donors. PBMC proliferation was determined by the effect of MSC to suppress anti-CD3/CD28-induced proliferation of PBMCs in a non-contact co-culture. Non-contact co-cultures of huMSC and PBMCs were prepared using transwell culture insert (0.4 µm, corning). HuMSC were seeded into 24-well plate at a concentration of 3*10^4^ cells per well to achieve the final PBMC:huMSC ratio 10:1. HuMSC were cultured for 24 hours (hrs) before the co-culture experiment. PBMCs were activated by anti-CD3 and anti-CD28 for 24 hrs and then labeled with CellTrace™ Violet Cell Proliferation Kit (Invitrogen). Transwell inserts with 3*10^5^ PBMCs per insert in 200 µl of FBS-supplemented RPMI-1640 culture medium were placed in huMSC-containing wells. After 5 days of co-culture, the proliferation of PBMCs was assessed by a flow cytometer (FACScan, BD Biosciences) and data were analyzed using FlowJo 7.6.1 software. Activated T cells alone without huMSC were used as a positive control.

**Immunocytochemistry**

For immunocytochemistry assay, we placed coverslips in culture well before seeding cells into the 24-well plate. After another 24-hour of culturing, cells were washed with PBS twice and fixed with 4% paraformaldehyde (PFA) for 20 minutes at room temperature. After this, cells were permeabilized with 0.1% Triton X-100 in PBS and blocked with 3% donkey serum in PBS. After incubation with the primary antibody anti-Nestin (1:1000, MAB5326, Abcam) at 4°C overnight, cells were then incubated with 568 Alexa Fluor-conjugated secondary antibodies (1:1000, A10037, Invitrogen) for 1 hr at room temperature. Finally, slides were mounted in Fluoromount G (SouthernBiotech, 0100-01) and visualized by fluorescence microscopy.

**Spinal cord injury (SCI) model**

C57BL/6 female mice, weighed about 20-25g were subjected to spinal cord crush injury with rush time being 3 seconds. 3 days after SCI, the animals were injected with control vehicle, 1 million huMSC/animal, through tail veins. HuMSC from three different donors with different suppressive indices (SI) were examined. BMS scoring of animal walking behavior was performed by observers blinded to treatment, for a period of 6 weeks.

**Table S1. Primers used in this study.**

| primer_name | primer (5'-3') |
| --- | --- |
| Human-IDO1-F | GCCCTTCAAGTGTTTCACCAA |
| Human-IDO1-R | CCAGCCAGACAAATATATGCGA |
| Human-CXCL9-F | GGTTCTGATTGGAGTGCAAGGA |
| Human-CXCL9-R | GGATAGTCCCTTGGTTGGTGCT |
| Human-IL6-F | ACTCACCTCTTCAGAACGAATTG |
| Human-IL6-R | CCATCTTTGGAAGGTTCAGGTTG |
| Human-GAPDH-F | AACCATGAGAAGTATGACAACAGC |
| Human-GAPDH-R | CATGTGGGGCCATGAGGTCCACCAC |
| Mouse-TNFα-F | GACCCTCACACTCAGATCATCTTCT |
| Mouse-TNFα-R | CCTCCACTTGGTGGTTTGCT |
| Mouse-GAPDH-F | GTATGACTCCACTCACGGCAAA |
| Mouse-GAPDH-R | GGTCTCGCTCCTGGAAGATG |

**Table S2.** Sample information of MSCs.

**Table S3.** BV2-inhibition correlated genes and GO enrichment analysis.

**Table S4.** Gene differential expression and GO enrichment analysis of 2F response genes.

**Table S5.** GSVA analysis of selected functions.

**Figure Legend**

**Figure S1.** Transcriptomic analyses revealed that differentially expressed genes from human passage 4-6 MSCs derived from different tissue sources (umbilical cord, fetal, adult bone marrow, adult adipose tissue) were involved in important biological processes including cell adhesion, cell proliferation, and etc.

**Figure S2**. (A) Different huMSC clones from the same donor demonstrated heterogeneity in that one clone was positive for Nestin and the other was negative. (B) These two clones demonstrated different efficacies in inhibiting human PBMC proliferations using T-cell proliferation assays. Left panel, negative control, middle panel, strong inhibition from CFU-13, and right panel, weak or almost no inhibition from CFU-15.

**Figure S3**. Different huMSC lines (A, SI=0.67; B, SI=0.52; C, SI=0.26) displayed variations in efficacies of treating spinal cord crush injury in mouse model. BMS scoring was used to evaluate motor behaviors of SCI mice in different groups. A cell line, but not B or C lines, was efficacious in improving animals motor behavior after SCI.

**Figure S4**. Semantic similarity network computation among GO terms enriched in genes that were positively correlated with BV2 inhibition capacity of huMSCs.

**Figure S5**. Semantic similarity network computation among GO terms enriched in genes that were up-regulated in huMSCs by 2-factor stimulation.

**Figure S6**. Semantic similarity network computation among GO terms enriched in genes that were down-regulated in huMSCs by 2-factor stimulation.
