# Supplementary figures and images for: Eradication of specific donor-dependent variations of mesenchymal stem cells in immunomodulation to enhance therapeutic values
Source: Cell Death Dis. 2021 Apr 6;12(4):357. doi: 10.1038/s41419-021-03644-5 (PMC8024246; doi:10.1038/s41419-021-03644-5)

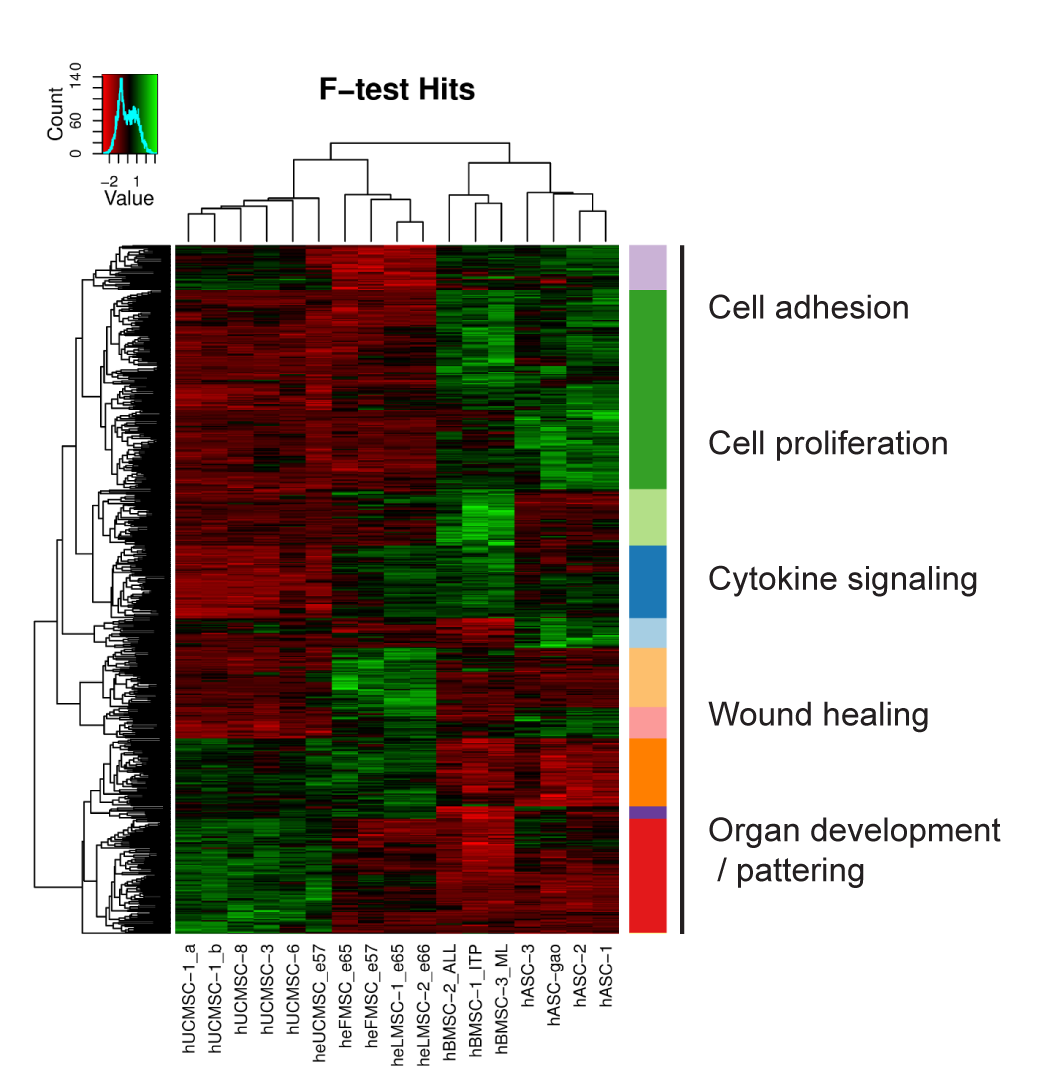

Supplement: Supplementary file 2 — Figure S1 [file 41419_2021_3644_MOESM2_ESM.tif]

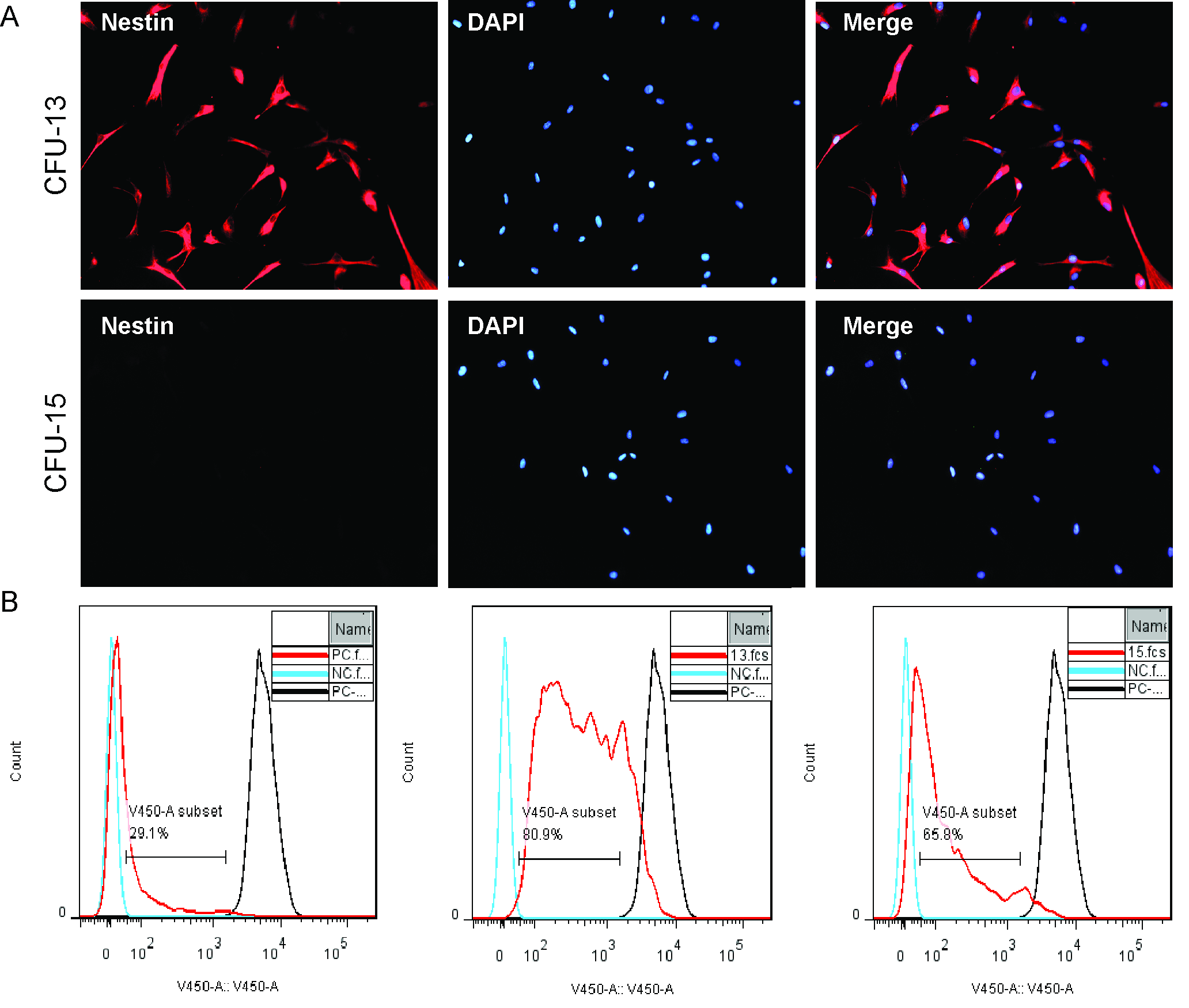

Supplement: Supplementary file 3 — Figure S2 [file 41419_2021_3644_MOESM3_ESM.tif]

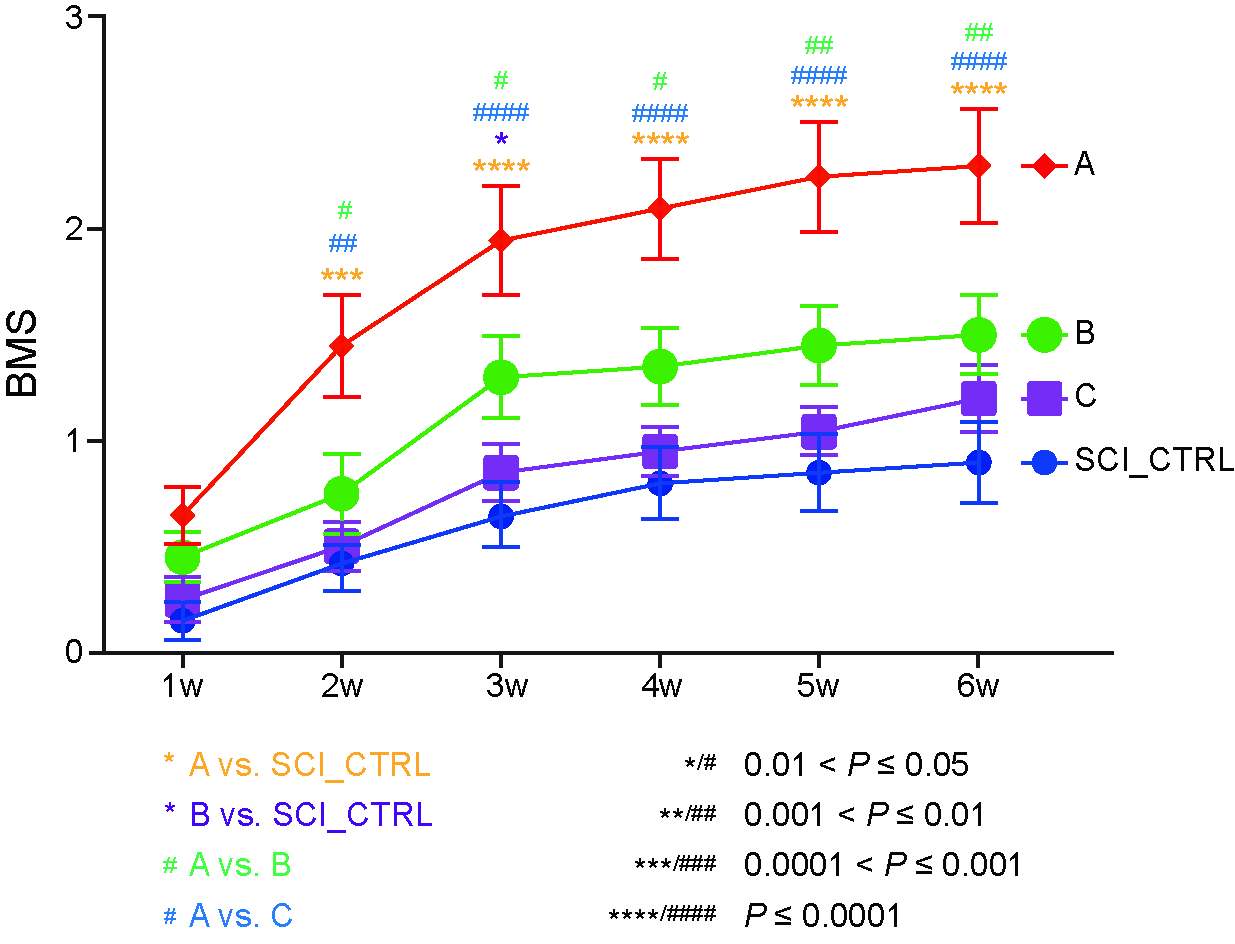

Supplement: Supplementary file 4 — Figure S3 [file 41419_2021_3644_MOESM4_ESM.tif]
